# Pathogenic aquaporin-4 reactive T cells are sufficient to induce mouse model of neuromyelitis optica

**DOI:** 10.1186/s40478-015-0207-1

**Published:** 2015-05-21

**Authors:** Melina V. Jones, Hwa Huang, Peter A. Calabresi, Michael Levy

**Affiliations:** Department of Neurology, Johns Hopkins University, 600 N. Wolfe Street, Pathology 509, Baltimore, MD 21287 USA

**Keywords:** Neuromyelitis optica, Aquaporin-4, Autoreactive T cells, Th17

## Abstract

**Introduction:**

Neuromyelitis Optica (NMO) is an autoimmune disease primarily targeting the spinal cord and optic nerve leading to paralysis and blindness. The discovery of an antibody against the astrocytic water channel, aquaporin-4 (AQP4), in the majority of patients, has led to the presumption that the antibody was necessary for disease pathogenesis. The potential role of T cells in the central nervous system, however, has not been thoroughly examined.

**Results:**

We generated an anti-AQP4 antibody seronegative model of NMO using pathogenic AQP4-reactive T cells in mice by immunizing AQP4 null mice with peptides corresponding to the second extracellular loop of AQP4, loop C. When polarized to a Th17 phenotype and transferred to wild-type mice, these cells caused tail and limb weakness. Histology showed demyelination and T cell infiltration in the spinal cord, optic nerve and brain. Animals receiving cells re-stimulated in culture with non-specific proteins resulted in no behavioral disease, indicating that specific targeting of AQP4 is essential for this phenotype.

**Conclusions:**

In summary, we show that AQP4-reactive T cells are sufficient to trigger an NMO-like disease in mice, independent of antibodies, indicating that pathogenic AQP4-reactive T cells may play a similar role in humans.

## Introduction

Neuromyelitis optica (NMO) is a relapsing autoimmune disease primarily targeting the spinal cord and optic nerve leading to paralysis and blindness [1]. The discovery of the highly specific anti-aquaporin-4 (AQP4) IgG_1_ biomarker implicates an immune reaction against AQP4 evidenced by both humoral and cellular pathology within acute NMO lesions [2, 3]. Several previous mouse and rat models of NMO focused on the role of the circulating anti-AQP4 antibody in disease pathogenesis concluded that the antibody by itself is insufficient to induce disease, but can exacerbate an experimental autoimmune encephalomyelitis induced by myelin-reactive T cells [4–8]. When the anti-AQP4 antibody has passive access to AQP4 on astrocytes in the nervous system, there is abundant evidence that the antibody binds to AQP4 and can participate in complement mediated damage to astrocytes under experimental conditions [9–11]. Collectively, these studies indicate an important role for the anti-AQP4 antibody in enhancing astrocytic damage from NMO relapses rather than in the triggering of NMO attacks, prompting the search for other AQP4-specific immune components that may be involved upstream in the immunopathogenesis of NMO. In each passive transfer study, the anti-AQP4 antibody was only pathogenic in the context of a T-cell based autoimmune attack on the central nervous system.

Production of the IgG_1_ biomarker against AQP4 presumably requires AQP4-reactive B and T cells for immunoglobulin class switching. T cells are also among the inflammatory cells found in acute NMO lesions and their role in the immunopathogenesis of this disease has been the subject of recent studies in which immunodominant AQP4 peptides can trigger T cell activation in mice [12, 13] . However, despite activating T cells against AQP4, these rat and mouse models do not develop a clinical neurological phenotype either because pathogenic T cells responses are limited by a combination of central and peripheral tolerance or because certain AQP4 epitopes are not pathogenic.

We used a unique approach to raise pathogenic AQP4-reactive T cells by immunizing AQP4 null mice with loop C peptide of AQP4 (AQP4_135–153_). When adoptively transferred into wild type mice after re-stimulation in culture with Aqp4_135–153_ peptide, behavioral disease was not induced though mild T cell infiltration of the central nervous system (CNS) was observed. Polarization of AQP4-reactive T cells to the T-helper-17 led to CNS inflammation characterized by demyelination and T cell infiltration into the spinal cord, optic nerve, and brain. There was no other evidence of solid organ inflammation despite widespread AQP4 expression in the mouse supporting the specificity of this approach to modeling the human NMO disease.

## Materials and methods

### Animals

Aquaporin-4 null mice, backcrossed onto the C57BL/6 background at least 14 times, were obtained from Erlend Nagelhus (University of Oslo, Oslo, Norway) and bred in-house. Female C57BL/6 wild-type mice between 6–8 weeks of age were purchased from The Jackson Laboratory. All mice were housed in a pathogen-free 12-h artificial light-dark cycle and had *ad libitum* access to food and water. The Johns Hopkins Institutional Animal Care and Use Committee approved all experimental procedures.

### T cell generation and culturing

Aquaporin-4 extracellular loop peptides (human 56–69, 135–53, and 212–30) were synthesized at the Johns Hopkins Synthesis & Sequencing Core Facility. Stock solutions of 120 mg/ml were prepared in DMSO. All three peptides were further diluted into phosphate-buffered saline each at 2 mg/ml and mixed 1:1 with complete adjuvant containing 8 mg/ml heat-killed *M. tuberculosis* H37Ra (Difco) in incomplete adjuvant (Imject; Thermo-Fisher) [14]. Aquaporin-4 KO and syngeneic C57Bl/6 mice (Jackson, MA, USA) were immunized in the flanks with a total of 100 μl of emulsion. Mice were also injected intraperitoneally with 250 ng of Pertussis toxin (Tocris) on days 0 and 2. Twenty-three days following immunization, spleens were harvested and single cell suspensions were prepared by pushing spleens through 70 μm cell strainers (Becton Dickenson) using syringe plungers. Red blood cells were depleted by resuspending each spleen in 2 ml of ACK lysis solution (Quality Biological, MD, USA) for 2 min at room temperature, followed by washing with media. Cells were counted and seeded in 96-well flat bottom plates at 3 × 10^5^ cells per well in RPMI 1640 supplemented with Glutamax, 1 % non-essential amino acids, 1 % sodium pyruvate, 1 % antibiotic-antimycotic (Life Technologies, Inc.), 10 % fetal calf serum (Sigma) and 50 μM betamercaptoethanol (Sigma). One hundred microliters of media containing peptide (final concentration: 10 μg/ml): MOG_35–55_, AQP4_56–69_ (loop A), AQP4_135–53_ (loop C), or AQP4_212–30_ (loop E) were added to wells in triplicate. Media with no peptide added or containing 0.1 % DMSO served as “no stimulus (NS)” background controls. After 4 days in culture in an incubator at 37 °C with 5 % CO_2_ in a humidified atmosphere, 10 μl of a solution containing 0.5μCu of ^3^H-thymidine (Perkin-elmer) were added to each well and incubated a further 18 h. Cells were harvested onto filter paper mat. After drying, mats were treated with scintillation fluid and assayed for ^3^H incorporation. Results are expressed as counts per minute (cpm).

### T cell polarization, adoptive transfer and behavioral scoring

Six to seven week old female aquaporin-4 KO mice were immunized with an emulsion of 4 mg/ml Complete Freund’s adjuvant with 1 mg/ml of Loop C peptide (135–53) on each flank and each shoulder (50 μl per injection site). Ten days following immunization, lymph nodes (inguinal, lumbar, brachial and axillary) were collected. Single cell suspensions were prepared by forcing nodes through a 70 μm cell strainer using a syringe plunger. After washing in complete media (RPMI 1640 with 10%FCS, 50 μM β-mercaptoethanol, non-essential amino acids, sodium pyruvate, HEPES), cells were counted and seeded into T75 flasks at 6 × 10^6^ cells per ml. Unpolarized cells were stimulated with peptide only (final concentration was 50 μg/ml). For Th17 polarization, cells were stimulated with 30 ng/ml IL-6, 20 ng/ml IL-23 (eBiosciences) and 10 μg/ml of anti-IFNgamma (XMG1.2; BD Biosciences) in addition to peptide. Following 3 days of culture, cells were harvested, washed with sterile PBS, and 5x10^6^ cells were injected into mice intravenously through their tail veins. Pertussis toxin (250 ng; Tocris) was injected intravenously immediately following cells and again two days later. Behavioral signs and weights were tracked starting 5 days post-transfer of cells, which was quantified using a standardized 5-point EAE disability scale by a blinded examiner [14]. Animals were euthanized and tissues harvested for histological evaluation 14–21 days following cell transfer.

### ELISPOT assay

ELISPOT assay was used to determine the frequency of cytokine-producing cells in polarized and unpolarized cell cultures. The day before cells were to be harvested from immunized AQP4 null mice, the wells of an immobilon P-bottom 96-well plates (Multiscreen®HTS, 0.45 μm pore size; EMB Millipore, USA) were pre-wet with 35 % ethanol for 30 s, washed three times with coating buffer, and coated with 50 μl of a 1:250 dilution of capture antibody, anti-IL17 (Th17), provided with ELISPOT Ready-Set-Go kits (eBioscience). Plates were covered and incubated overnight at 4 °C. Wells were then washed twice with coating buffer and once with complete media; plates were stored in the incubator until cells were prepared. Spleen and lymph node cells were prepared as described above and prepared with and without polarizing conditions. Harvested cells were 2-fold serially diluted in media containing 3 × 10^7^ irradiated splenocytes (irradiated with 3,500 rads) as antigen-presenting cells (APCs); APCs mixed 12:1 with 1.25 × 10^5^ lymph node cells per well yielded the most well-defined spots. After overnight in culture, wells were washed (TBS + 0.05 % Tween®-20). Detection antibody was diluted 1:250 in diluents provided in the kit and 50 μl applied to each well for 2 h at RT. After washing, streptavidin-alkaline phosphatase was added to each well (1:2500; Sigma) for 45 min. After further washing, signal was developed with development solution containing BCIP and NBT (i.e. 150 mM Tris-HCl, 5 mM MgCl^2^, 100 mM NaCl, pH9.5 supplemented with 4 mM levamisole (Sigma), 0.15 mg/ml 5-bromo-4-chloro-3-indolyl-phosphate, and 0.36 mg/ml 4-nitro blue tetrazolium chloride (Roche) for 10 min at room temperature in the dark. Reaction was stopped by washing with PBS then distilled H^2^O before air drying. Spots were imaged with an Immunospot Series 3 Analyzer (Cellular Technology, Ltd.) and counted using Image J and the “find Maxima” function. Results are expressed as the mean of triplicate values (± standard error of the mean, SEM) adjusted to per 10^6^ cells per well. Student’s *t*-test was performed on data; p<0.05 was considered statistically significant.

### Tissue processing and histology

Animals were anesthetized with isofluorane and perfused via cardiac puncture first with PBS and then with freshly prepared 4 % paraformaldehyde solution. The optic nerves and spinal cords were harvested, fixed overnight, cryopreserved in 30 % sucrose and frozen for sectioning. After embedding tissue in O.C.T. Compound (Tissue-Tek®), ten micron slices sections were mounted on Superfrost Plus Microscope Slides (Fisher brand). Eriochrome cyanine was used to identify demyelinating lesions in the sectioned tissue. Eriochrome cyanine solution was prepared by dissolving eriochrome cyanine in 450 ml 0.5 % H_2_SO_4_ (0.2 %) and 10 % FeCl_3_ added to a final concentration of 0.4 %. The sectioned tissue was hydrated by serial washes in 100 % ethanol, 95 % ethanol, 70 % ethanol and distilled water for 10 min each and then immersed for 15 min in eriochrome cyanine solution. After staining, differentiation was carried out in freshly made 0.1 % NH_4_OH for 20–30 s and halted by thorough washing in distilled water. Slides were mounted as described below. Sections were counterstained with 0.1 % eosin Y in acetate buffer. Immunohistochemical staining for CD3+ T cells was performed by washing sections in saline before performing heat-mediated antigen retrieval in 0.05 M sodium borate buffer (pH 8.0) in a microwave pressure cooker. Slides immersed in buffer were heated in the microwave at full power until full pressure was achieved (5 min) then heated for an additional 7 min at 20 % power. After 3 min of cooling and flooding of slide container with room temperature saline, slides were transferred to 3 % H_2_O_2_ for 20 min to quench endogenous peroxidases and blocked for endogenous biotin using an avidin/biotin blocking kit (Vector Laboratories, Inc.). Non-specific binding was blocked with 5 % goat serum in 0.1 % Triton®X-100 for 30 min at room temperature. Anti-CD3 rabbit monoclonal antibody (clone SP7; GeneTex,USA) was applied at 1:75 overnight at 4 °C and detected with biotinylated goat anti-rabbit IgG (1:1000; Vector Laboratories, Inc.), followed by Avidin-Biotin Complex-horse radish peroxidase (Vector Laboratories, Inc.). Signal was developed with 0.5 mg/ml diaminobenzidine HCl in PBS with 0.03 % H_2_O_2_ for 5 min. After washing, slides were Fast Green counterstained, dehydrated and mounted. Quantification and analysis of myelin and CD3 staining was as described [7]. Glial fibrillary acidic protein (GFAP), myelin basic protein for myelin, and aquaporin-4 were examined by immunofluorescence (without antigen retrieval) applying mouse anti-GFAP (1:1000; Sigma), rabbit monoclonal anti-MBP (1:250; Epitomics/ Abcam), and rabbit anti-AQP4 (H-19) (1:250; Santa Cruz Biotechnology) overnight at 4 °C, followed by goat Alexa Fluor® 555-conjugated anti-rabbit IgG and Alexa Fluor® 488-conjugated anti-mouse IgG (1:250; Life Technologies / Molecular Probes) for 30 min at RT. Fluorescent sections were mounted with Fluorogel (Electron Microscopy Sciences) containing 2 μg/ml 4′,6-Diamidino-2-Phenylindole (DAPI) and sealed with clear nail polish.

CD3 quantification was performed in a blinded fashion using background-corrected high resolution images from areas representing 6–8 levels of the spinal cord, 3 coronal levels of the cerebrum (left and right, for a total of 6 fields of view captured at 4X), or a region from the left and right lobes of the cerebellum (2 fields at 4X) via Fast Green staining. Three sections from each optic nerve were also analyzed (each representing a part of the longitudinal length of the nerves). Total immunoreactive (brown DAB reaction product) was expressed as % immunoreactive area and mean ± SEM.

## Results

**Fig. 1:**
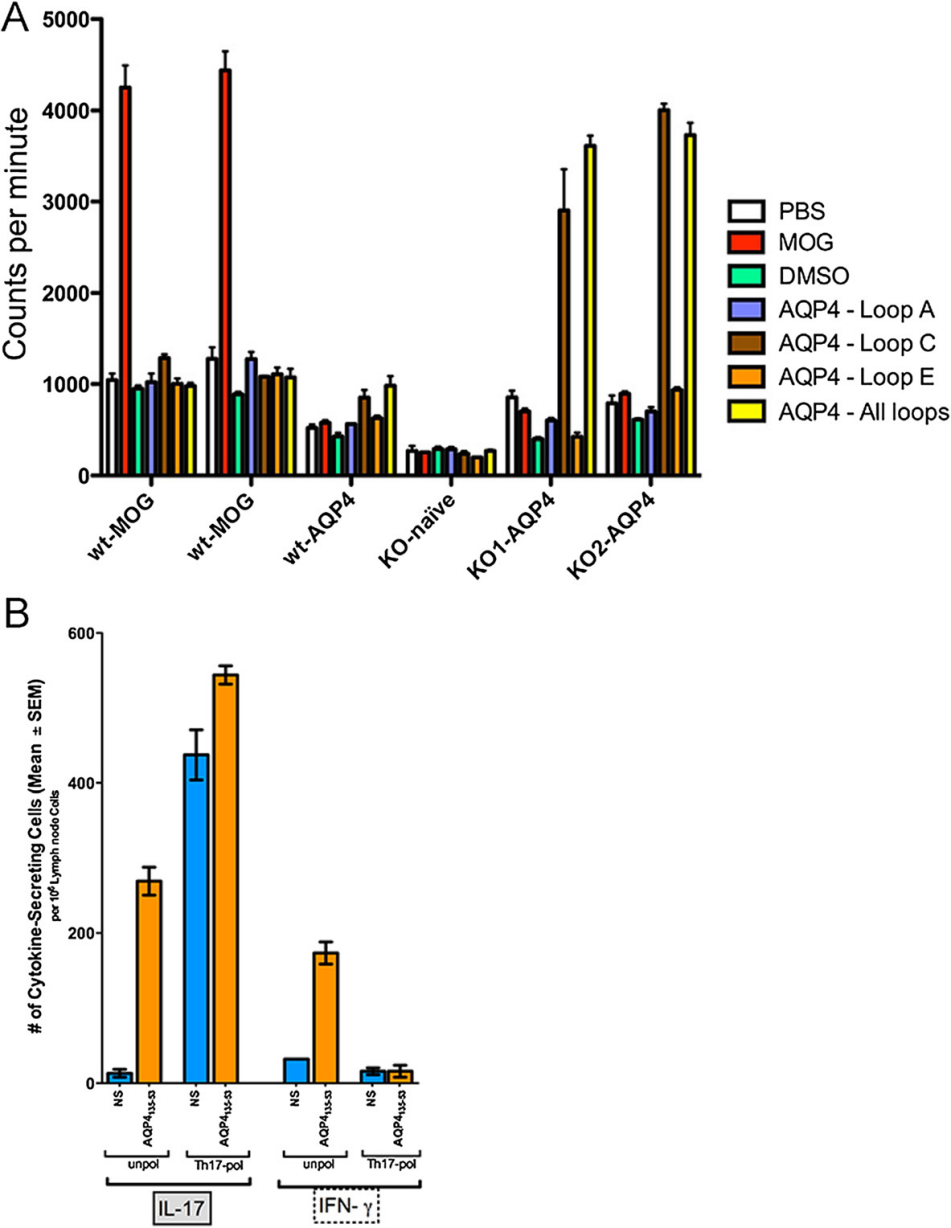
**A**. T cell proliferation assay reflecting a robust reaction to loop C of AQP4. T cells from two C57Bl6 mice (wt-MOG) immunized with myelin-oligodendrocyte glycoprotein (MOG) peptide 35–55 showed proliferative activity as measured by incorporation of tritiated-thymidine only in the presence of the MOG antigen (red bars). C57Bl6 mice immunized with AQP4 peptides (wt-AQP4) did not react against any AQP4 antigen. T cells from AQP4 null mice (KO naïve) do not inherently react to AQP4 peptides unless the mice are immunized with the peptides (KO1-AQP4 and KO2-AQP4). Among those tested, a peptide corresponding to the extracellular loop C generated a robust reaction only in T cells from AQP4 null mice immunized against a mix of AQP4 peptides 56–69 (loop A), 135–53 (loop C), and 212–30 (loop E), exposed to cells in culture alone (brown bars) or as a mixture of all three peptide (yellow bars). Results shown are counts per minute means +/− SEM of triplicate reactions. **B**. ELISPOT assay was used to determine the number of IL-17 and interferon-gamma (IFN-γ) cytokine-producing cells in Th17 polarized (Th17-pol) and unpolarized (unpol) cell cultures exposed to AQP4 loop C peptide (AQP4_135–53_) versus no stimulation (NS). Unpolarized AQP4-reactive T cells expressed significant levels of both IL-17 and IFN-γ compared to unstimulated controls. After polarization to a Th17 phenotype, the number of IL-17 producing cells almost doubles while the number of IFN-γ producing cells is nearly undetectable, but the frequency of IL-17-producing cells also increases in the unstimulated culture (while IFN-γ-producing cells remains low)

In NMO, the anti-AQP4 antibody targets an extracellular epitope of AQP4 [15]. Using AQP4 null mice, we checked for antibody production and T cell responses against peptides corresponding to the extracellular loops of AQP4, loops A, C and E. AQP4 null mice were used in this model because these mice do not have to overcome immune tolerance to develop pathogenic AQP4-reactive T cells. We found no evidence of antibody production against any of the loops. The lack of anti-AQP4 antibody production is not unexpected as short peptides do not routinely exhibit robust antibody responses. However, there was a robust T cell response against the second extracellular loop of AQP4, loop C (Fig. 1a). In phenotyping these AQP4-reactive T cells, we found that a significant number of these unpolarized cells produced both interleukin-17 (IL17) and IFN-gamma, compared to non-stimulus (NS) controls (Fig. 1b). Polarization of T cells towards the Th17 phenotype (Th17-pol) produced twice the number of IL17-secreting cells while the number of IFN-gamma producing cells were barely detectable (Fig. 1b).

**Fig. 2:**
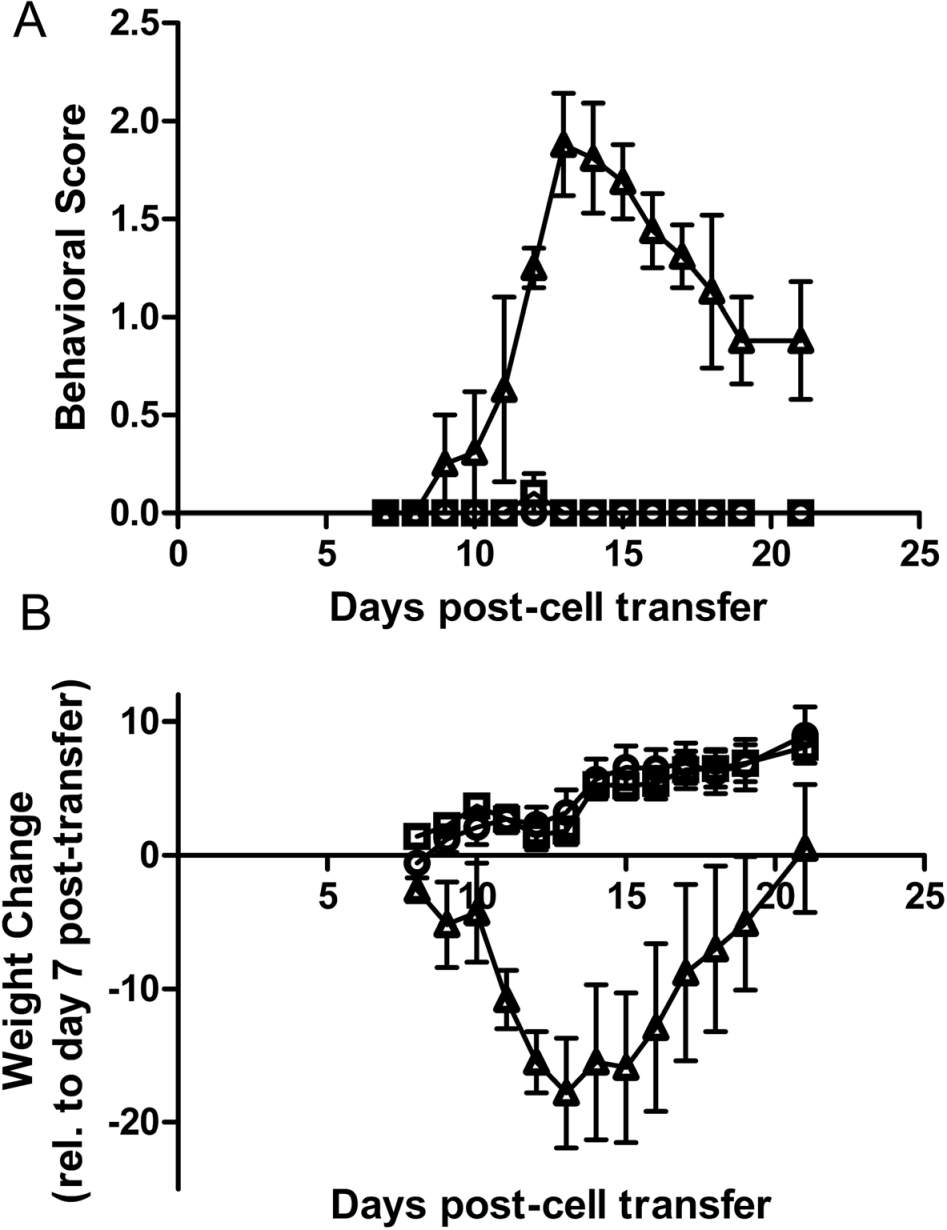
**A**. Behavioral assessment of adoptive intravenous transfer of T cells from AQP4 null mice immunized against loop C peptide of AQP4. The Behavior Score is a 5 point EAE-scale scoring the extent of neurologic disability (0 is no disability, 5 is death). Wildtype mice (triangles) adoptively transferred with cultured AQP4-restimulated, Th17 polarized AQP4-reactive T cells develop weakness in the tail and hind limbs (EAE score of 1.0–2.0, n = 4). Transfer of AQP4-reactive T cells that are not re-stimulated with AQP4 peptide (squares, n = 5) or stimulated with non-specific proteins (circles, n = 6) show no behavioral phenotype. **B**. Daily weights show typical weight loss in mice receiving the Th17 polarized AQP4-reactive and AQP4-restimulated T cells (triangles), but not in mice that receive unstimulated (circles) or non-AQP4-specifically re-stimulated T cells (squares)

The AQP4 null mice that generated a robust T cell reaction against the second extracellular loop of AQP4, loop C, did not develop autoimmune neurological disease as they do not express the target antigen. Even when intravenously transferred to wildtype AQP4-expressing C57Bl/6 mice, these AQP4-reactive T cells did not develop clinically meaningful behavioral manifestations or histological evidence of CNS demyelinating disease beyond meningeal inflammation (data not shown). Prior to adoptive transfer, when AQP4-reactive T cells were polarized to a stronger pro-inflammatory Th17 helper cell phenotype, the clinical effect was dramatic with leg weakness and paralysis in addition to a drooping tail with behavioral scores of at least 2.0 (Fig. 2) and associated weight loss.

Three important controls showed no clinical or histological phenotype, confirming the specificity of this model. Adoptive transfer of Th17 polarized AQP4-reactive T cells from a. unstimulated cultures or b. cultures stimulated with non-specific proteins was harmless highlighting the requirement of AQP4-peptide during the polarization process (Fig. 2). Pathogenic AQP4-reactive T cells polarized to Th17 that were transferred back to naïve AQP4 null mice is also harmless demonstrating that astrocytic expression of AQP4 in the host mouse is necessary for this model (data not shown).

**Fig. 3:**
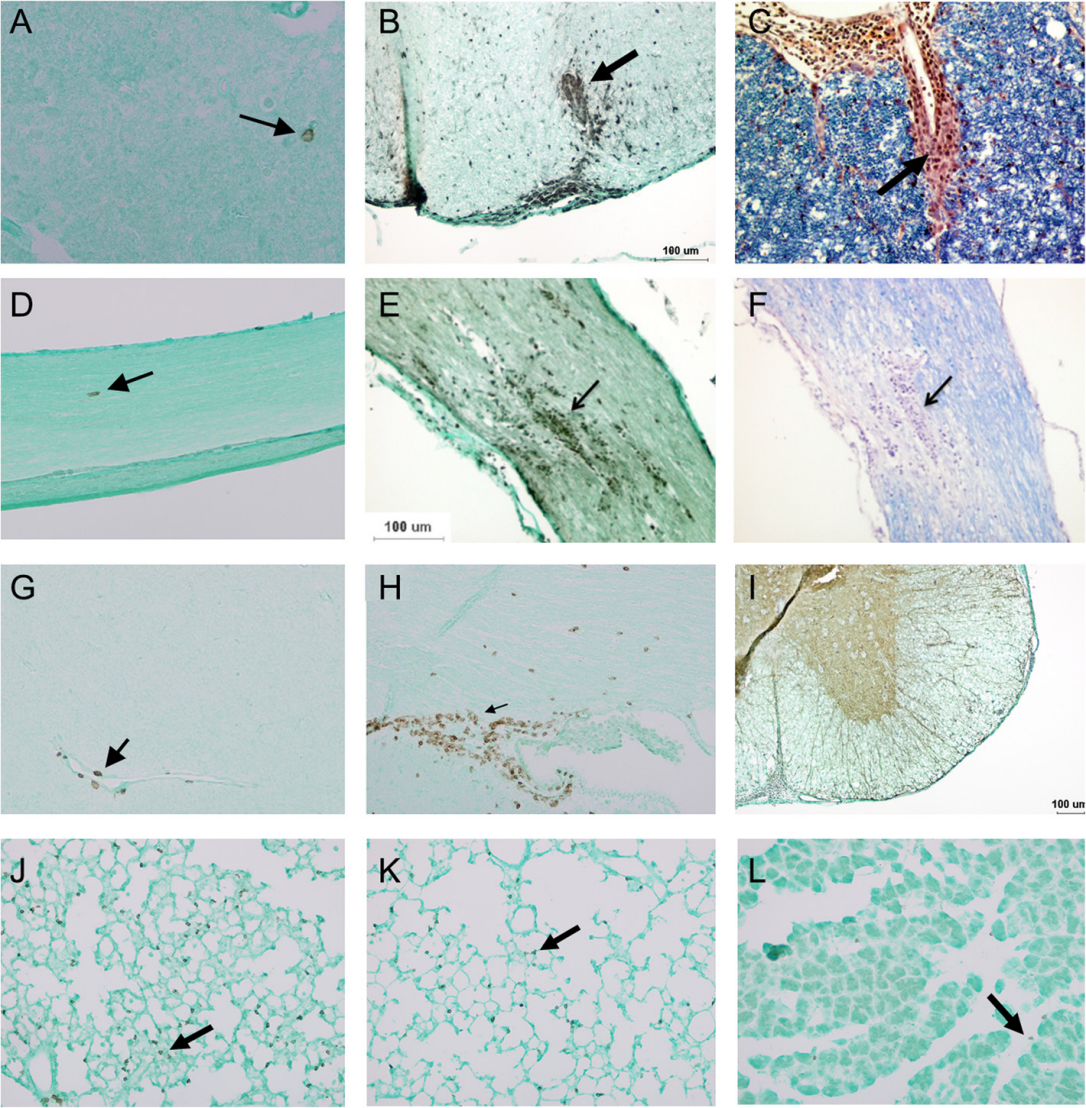
Histology of tissues from wildtype mice receiving unpolarized AQP4-reactive T cells **(A, D, G, J)** versus wildtype mice receiving Th17-polarized AQP4-reactive T cells **(B, C, E, F, H, I, K, L)**. **A**. Spinal cord parenchyma stained for CD3+ T cells shows rare, scattered cell (arrow), compared to **B**. spinal cord sections from wildtype recipients of Th17-polarized AQP4-reactive T cells which shows intense perivascular CD3+ T cell infiltrates. **C**. Areas of demyelination (red) within white matter tracts (blue) are visible within inflammatory lesions. **D**. Longitudinal sections of optic nerves stained for CD3+ T cells shows rare, scattered cell (arrow), compared to **E**. optic nerve sections from wildtype recipients of Th17-polarized AQP4-reactive T cells which shows intense perivascular CD3+ T cell infiltrates (arrow) **F**. Areas of demyelination (arrow pointing to red) within white matter tracts (blue) are visible within inflammatory lesions. **G**. Brain parenchyma stained for CD3+ T cells shows rare, scattered cell (arrow), compared to **H**. a brain section from wildtype recipients of Th17-polarized AQP4-reactive T cells which shows intense CD3+ T cell infiltrates, such as this lesion around the 3^rd^ ventricle (arrow). **I**. AQP4-reactive T cells do not appear to change AQP4 staining either in lesions or in normal appearing spinal cord, optic nerve or brain despite widespread inflammation and demyelination (representative section from spinal cord shown). **J**. Despite expression of AQP4 in solid organs, rare AQP4-reactive CD3+ T cells appear scattered throughout these organs both in the unpolarized and Th17-polarized wildtype recipients. Lung from unpolarized shown here with arrow pointing to CD3+ cells. **K**. Lung section from Th17-polarized recipient showing normal lung with occasional CD3+ cells (arrow). **L**. Muscle from Th17 polarized mice show no evidence of inflammation (arrow pointing to rare CD3+ T cells)

**Fig. 4:**
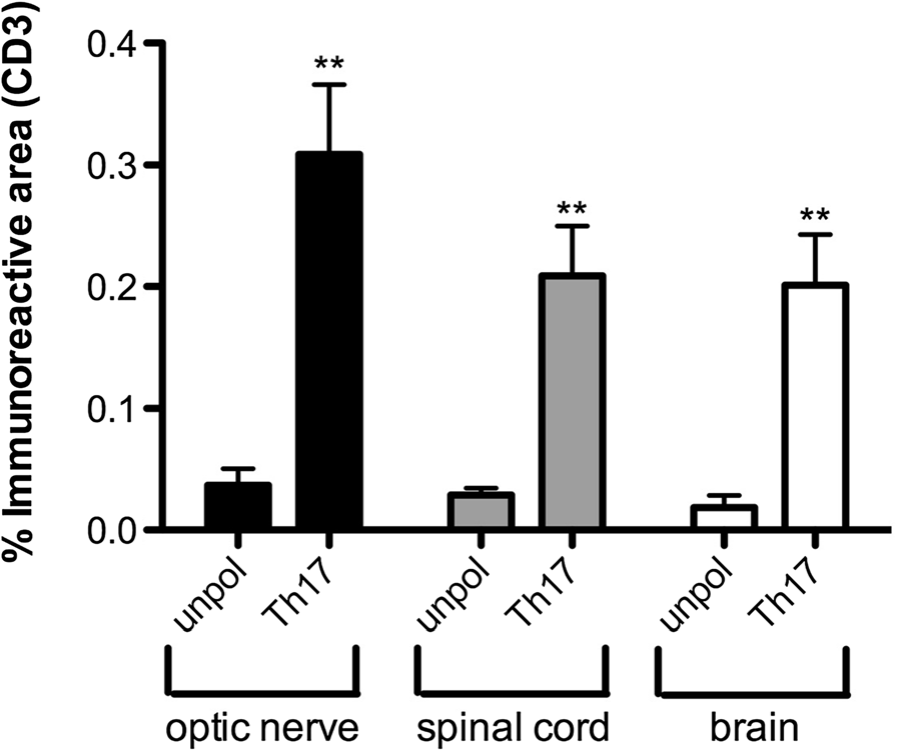
Blinded quantification of CD3 cells in the spinal cord (n = 8), optic nerve (n = 6) and brain (n = 8) of wildtype recipients of Th17-polarized AQP4-reactive T cells shows >5-fold more immunoreactivity (**p<0.01) in these tissues compared to unpolarized mice

Histologically, in clinically asymptomatic wildtype recipients of unpolarized AQP4-reactive T cells, there were rare CD3+ T cells scattered in the parenchyma of the spinal cord, optic nerve and brain (Fig. 3a, d, g), as well as other AQP4-expressing solid organs such as the lung (Fig. 3j). In clinically-affected wildtype recipients of adoptively transferred Th17-polarized AQP4-reactive T cells, histology revealed demyelination and increased inflammatory infiltrate comprised primarily of CD3+ lymphocytes in the spinal cord, optic nerve and brain. Inflammation and demyelination in the spinal cord (Fig. 3b, c) and optic nerves (Fig. 3e, f) accounted for the majority of symptoms, but parts of the brainstem, cerebellum and cerebral cortex showed areas of inflammation that were not as clinically obvious in the mice (Fig. 3h). Although the AQP4 water channel is targeted by these pathogenic T cells, astrocytic AQP4 expression appears relatively intact even within acute inflammatory lesions (Fig. 3i). Despite AQP4 expression in many other solid organs, there is no evidence of inflammation or AQP4 loss outside of the CNS including the lung (Fig. 3k) or muscles (Fig. 3L) in clinically affected mice. Blinded quantification of CD3 cells in the spinal cord, optic nerve, and brain of wildtype recipients of Th17-polarized AQP4-reactive T cells shows >5-fold more immunoreactivity (**p<0.01) compared to mice that received unpolarized AQP4-reactive T cells (Fig. 4).

## Discussion

This study demonstrates that adoptive intravenous transfer of pathogenic AQP4-reactive T cells is sufficient to cause an NMO-like inflammatory disease that attacks the optic nerves, spinal cord and brain while sparing other AQP4-expressing non-CNS organs. Polarization of AQP4-reactive T cells to a stronger pro-inflammatory Th17 phenotype prior to transfer amplifies the inflammation leading to more severe demyelination and neurological dysfunction. The best explanation for this response is that immunization with AQP4-loop C peptide in AQP4 null mice generates a T cell response that is different from immunization of the same loop in wildtype mice [16]. The AQP4-reactive T cells in null mice are not exposed to any degree of negative selection as they likely would be if raised in wildtype mice. There may also be a protective regulatory response in wildtype mice that suppresses any potential auto-reactive tendency.

The implications of this study point to a key immunopathogenic role of Th17 polarized AQP4-reactive T cells of NMO in both triggering and localizing inflammation to AQP4 within the central nervous system. Pathogenic T cells targeting AQP4 do not kill AQP4-expressing astrocytes and do not cause loss of AQP4 expression. Rather, their role as demonstrated in this model is to trigger attacks directed towards AQP4-rich areas of the CNS and then recruit other components of the immune system, including antibodies and complement, to mediate the astrocytic damage. In human NMO pathology, death of astrocytes and loss of aquaporin-4 is a more downstream event initiated with binding of anti-AQP4 antibody, which leads to either complement-mediated destruction of the M23 isoform or internalization of the M1 isoform of AQP4 [17, 18]. We and others have previously demonstrated a pathogenic function of the anti-AQP4 antibody in exacerbating neuroinflammation in rodents, but not in instigating the disease [5–7]. The specificity of the immune target to the nervous system is not mediated by the anti-AQP4 antibody as it will bind AQP4 in any organ [19].

Interestingly, the extracellular loop C is the most common target of the anti-AQP4 antibody in humans [15]. AQP4-reactive T cells and a pathogenic anti-AQP4 antibody may work together to cause NMO. In this model, a susceptible person is exposed to a peptide corresponding to loop C of AQP4 under conditions that stimulate both an auto-reactive T cell and antibody reaction. A pro-inflammatory Th17 response to AQP4 may cause a more fulminant disease as demonstrated in this study and previous animal models [20]. Only after AQP4-reactive T cells trigger inflammation directed to the optic nerves and spinal cord would anti-AQP4 antibodies exacerbate the pathology by fueling complement activation and granulocyte recruitment.

This model highlights the potential for AQP4-specific immunotherapy for NMO. As a disease with a highly specific antigen (AQP4) and antibody response (anti-AQP4) associated with AQP4-reactive T cells, NMO is poised for treatment with an antigen-specific therapy [21, 22]. To induce a tolerance response, high dose soluble loop C peptide may be provided to patients in the setting of immunosuppression commonly used to treat NMO currently. With pre-existing disease, an oral route to achieve mucosal tolerance may be the safest initial approach to avoid worsening the disease [23]. In other diseases with less antigen-specificity, such as rheumatoid arthritis and multiple sclerosis, there is significant heterogeneity in the immunodominant antigen responses; in contrast, NMO is defined by reaction primarily to the AQP4 water channel although the precise target within AQP4 may vary slightly among NMO patients with some patients producing antibody responses against loops A and E as well [19]. A study in Lewis rats in which T cells reactive against loop E could induce inflammation in the spinal cord suggests that extracellular targets of AQP4 other than loop C may be involved [24].

## Conclusions

This model demonstrates the potential of AQP4-reactive T cells to contribute to behavioral disease and to oligodendrocyte damage in the central nervous system, independent of their peripheral role in generating high affinity astrocytoxic antibodies. New therapeutic options targeting such Th17 AQP4-reactive T cell in patients suffering from NMO could be examined in this model. Further study is required to determine how oligodendrocytes are killed by T cells reactive to an astrocyte protein. Indeed, how Th1 and Th17 T cells indirectly contribute to cellular damage through direct interactions with the antigen-presenting cells remains an unanswered question in NMO and multiple sclerosis pathogenesis. While antibody activation of complement and Fc-receptor-mediated cytotoxicity are well-established as important mediators in NMO pathology, this model provides a tool for examining T cell-mediated antibody-independent pathways in disease pathogenesis.
